# Phytochemical analysis, antiproliferative and antioxidant activities of *Chrozophora tinctoria*: a natural dye plant

**DOI:** 10.1080/13880209.2016.1277767

**Published:** 2017-02-05

**Authors:** Feyza Oke-Altuntas, Selma Ipekcioglu, Ayse Sahin Yaglioglu, Lutfi Behcet, Ibrahim Demirtas

**Affiliations:** a Department of Biology, Faculty of Science, Gazi University, Ankara, Turkey;; b Department of Chemistry, Faculty of Science, Cankiri Karatekin University, Cankiri, Turkey;; c Department of Biology, Faculty of Science Bingol University, Bingol, Turkey

**Keywords:** HeLa, HPLC/TOF-MS, radical scavenging, dyer’s croton, BrdU ELISA

## Abstract

**Context:***Chrozophora tinctoria* (L.) A. Juss. (Euphorbiaceae) is known as ‘dyer’s-croton’ and used to obtain dye substances. Recently, natural antioxidants and colorants have been of interest because of their safety and therapeutic effects.

**Objective:** This study investigates the antiproliferative and antioxidant activities of the various extracts and fractions from *C. tinctoria* and analyzes their phytochemical contents.

**Materials and methods:** The aerial parts of *C. tinctoria* were extracted with water, ethyl acetate, *n*-butanol, and methanol/chloroform. Phenolic compounds and other constituents of the extracts were analyzed by HPLC/TOF-MS. The ethyl acetate extract (EA) was fractionated by flash chromatography. The extracts, fractions, and major phenolic compounds were investigated for their antiproliferative activities on human cervical adenocarcinoma (HeLa) cell line at the concentrations of 5–100 μg/mL by using BrdU ELISA assay during 24 h of incubation. DPPH radical scavenging activities (5–150 μg/mL) and total phenolic contents of the samples were also evaluated.

**Results:** 4-Hydroxybenzoic acid (268.20 mg/kg), apigenin-7-glucoside (133.34 mg/kg), and gallic acid (68.92 mg/kg) were the major components of EA. CT/E-F6 (IC_50_ = 64.59 ± 0.01 μg/mL) exhibited the highest antiproliferative activity. CT/E-F2 (IC_50_= 14.0 ± 0.0 μg/mL) and some fractions displayed higher radical scavenging activity compared to synthetic antioxidant BHT (IC_50 _=_ _23.1 ± 0.0 μg/mL). Among the main phenolics, gallic acid exhibited the highest antiproliferative and radical scavenging abilities (IC_50 _<_ _5 μg/mL).

**Conclusion:** In this study, we have determined the biologically active fractions and their high effects may be attributed to the presence of gallic acid.

## Introduction

The genus *Chrozophora* Neck. ex A.Juss. (Euphorbiaceae) contains eight species. *Chrozophora tinctoria* (L.) A. Juss. is known as ‘Akbaş (white head)’ (Baslar & Mert 1999), ‘dyer’s-croton’, ‘giradol’ or ‘turnsole’ (Delazar et al. 2006). The plant is used to obtain dye substances (Guarrera 2006) including three main colors; red, yellow, and blue (Başlar 2000). *C. tinctoria* a produces dark blue dye due to its high solubility in water (Ugulu et al. 2009) and flavonoid content (Hashim et al. 1990). The plant is used traditionally to treat warts, and it has also been used as an emetic and cathartic and for the treatment of fever (Delazar et al. 2006).

Plants have always played an important role as a source of drugs (Shakhatreh 2013). Recently, natural antioxidants and colorants present in foods have attracted interest because of their safety and potential nutritional and therapeutic effect (Espín et al. 2000). Several synthetic dyes have been banned because they cause allergy-like symptoms or are carcinogens. Moreover, naturally derived colorants are also used in the cosmetic industry due to lack of side effects, UV protection, and anti-aging properties (Chengaiah et al. 2010).

The bioassay guided fractionation of this plant species has not previously been reported. The aim of this study was (i) to evaluate antiproliferative activities of the various extracts against HeLa cell line by BrdU Elisa assay (ii) to analyze the phytochemicals of the extracts by HPLC-TOF/MS, (iii) to fractionate the active extract by flash chromatography, (iv) to investigate antiproliferative and antioxidant activities of these fractions by using complementary assays.

## Materials and methods

### Chemicals and reagents

Anhydrous sodium carbonate, Folin–Ciocalteu’s phenol reagent, ethyl acetate (HPLC gradient grade), methanol (analytical reagent and HPLC gradient grade), 1-butanol, chloroform were purchased from Merck (Darmstadt, Germany). 2,2-diphenyl-1-picrylhydrazyl (DPPH), gallic acid, 2,6-di-*tert*-butyl-4-methylphenol (BHT), phenolic standards, formic acid, fetal bovine serum (FBS), penicillin/streptomycin, dimethyl sulphoxide (DMSO), Dulbecco’s modified Eagle’s medium-high glucose (DMEM-HG), gallic acid, 4-hydroxybenzoic acid, apigenin-7-glucoside, and 5-fluorouracil were purchased from Sigma-Aldrich GmbH. (Taufkirchen, Germany). All solvents used in HPLC analysis were HPLC grade and purchased from Merck.

### Plant materials

*Chrozophora tinctoria* materials were collected from Karali village (Elazığ-Turkey) in August 8, 2012 (20 km from Elazığ to Baskil, west of Karali village, fields, 1000-1200 m). The identification of plant materials were confirmed by taxonomist in the Department of Biology, Bingol University, Turkey. A voucher specimen (Behçet 8259b) was deposited at the Herbarium of the Biology Department, Bingol University, Turkey.

### Preparation of plant extracts

The dried aerial parts of *C. tinctoria* were powdered in a mill. For water extraction, the plant material (50 g) was boiled with 1 L of distilled water for 2 h and filtered through Whatman No. 1 filter paper. The aqueous extract was subsequently extracted with ethyl acetate (EA) and *n*-butanol (n-BuOH). The plant residue was finally extracted with 1:1 (v/v) methanol/chloroform (MeCh). The organic layers of EA, n-BuOH and MeCh were concentrated under reduced pressure.

### Fractionation of the ethyl acetate extract by flash chromatography

The ethyl acetate extract (10 g) was dissolved in methanol (20 mL, HPLC grade) and mixed with silica gel (10 g). The solvent was evaporated and the solid silica-extract mixture was subjected to flash chromatography column (Combiflash^®^ Rf, Teledyne Isco, Lincoln, NE) using hexane, CHCl_3_-hexane (1:1, v/v) and methanol. Fractions were combined with TLC (using ethyl acetate-methanol solvent system (1:1, v/v)) visualized under UV light (254 nm) and cerium (IV) sulfate (2 g cerium (IV) sulfate was dissolved in 100 mL of 15% H_2_SO_4_).

### HPLC-TOF/MS analysis

Phenolic compounds of the extracts were quantified by using Agilent 1260 Infinity HPLC system (Agilent, Santa Clara, CA) coupled with an Agilent 6210 TOF-MS detector and an Agilent Zorbax SB-C18 column (100 mm ×4.6 mm 3.5 μm). Mobile phases A and B were water/1 mL L^−1^ formic acid and acetonitrile, respectively. The flow rate was 0.8 mL/min, column temperature was 35 °C, and injection volume was 5 μL. The elution program was as follows: 0–1 min, 10% B; 1–12 min, 40% B; 12–14 min, 90% B; 14–17 min, 90% B; 17–18 min, 10% B; 18–25 min, 10% B.

### Antiproliferative effect by BrdU ELISA assay

The tested samples and 5-fluorouracil (5-FU; positive control) were dissolved in dimethyl sulfoxide (DMSO). Then the stock solution was diluted with Dulbecco’s modified eagle medium (DMEM). DMSO concentration was below 0.1% in stock solutions. HeLa cell line was grown in DMEM supplemented with 10% of fetal bovine serum (FBS) and 2% of penicillin-streptomycin. The medium was changed twice a week.

The extracts, fractions, and major phenolic compounds were investigated for their antiproliferative activities against HeLa cell line by using BrdU ELISA assay (Ceyhan et al. 2013; Karakus et al. 2013). Cultured cells were grown in 96-well plates (COSTAR, Corning, NY) at a density of 3 × 10^4^ cells/well. In each experimental set, the cells were plated in triplicates and replicated twice. The cell lines were exposed to eight concentrations of the samples and 5-FU for 24 h at 37 °C in a humidified atmosphere of 5% CO_2_. The cells were than incubated for overnight before applying the BrdU Cell proliferation ELISA assay reagent (Roche, Germany), according to manufacturer’s procedure. The amount of cell proliferation was assessed by determining the A450 nm of the culture media after addition of the substrate solution by using a microplate reader (Awareness Chromate, Palm City, FL). Results were reported as percentage of the inhibition of cell proliferation, where the optical density measured from vehicle-treated cells was considered to be 100% of proliferation. All assays were repeated twice. Percentage of inhibition of cell proliferation was calculated as follows: (1- A treatments/A vehicle control) × 100. Differences between groups were determined by ANOVA method (*p* < 0.01). IC_50_ value was determined using ED50plus vol. 1 software (developed by Mario H. Vargas, Bethesda, MD). 

### DPPH radical scavenging assay

Free radical scavenging activity of the samples was measured using the 2,2-diphenyl-1-picrylhydrazyl (DPPH) according to method of Blois (1958). The sample solutions were added to 0.004% methanol solution of DPPH. The mixture was shaken vigorously and left to stand at room temperature for 30 min in the dark. The absorbance was measured at 517 nm against a blank by a spectrophotometer (Rayleigh UV-2601, BRAIC Co. Ltd., Beijing, China). Scavenging of DPPH radical was calculated according to the formula: Scavenging % = [(*A*_control_–*A*_sample_)/*A*_control_] × 100. BHT was used as a positive control.

### Determination of total phenolic contents

Total phenolic contents of the extracts were determined using the modified Folin–Ciocalteu method as described by Singleton and Rossi (1965). The sample solutions were mixed with 0.2 mL of 50% Folin–Ciocalteu reagent and allowed to react for 3 min and 1 mL of aqueous solution of 2% Na_2_CO_3_ was added. At the end of the 45 min incubation at room temperature, absorbance of each mixture was measured at 760 nm. The same procedure was also applied to the standard solutions of gallic acid. Total phenolic contents were expressed as μg gallic acid equivalents per mg of the fractions/extract.

## Results and discussion

### Phytochemical analysis

**Figure 1.**
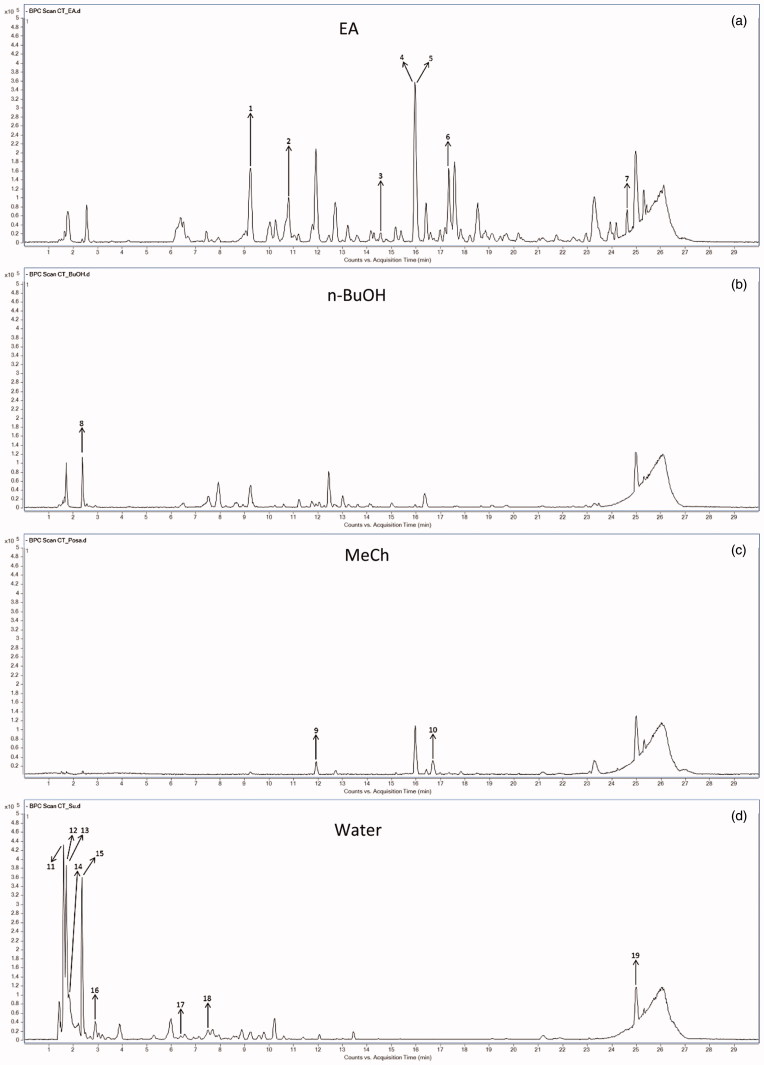
HPLC-TOF/MS chromatograms of the extracts from *C. tinctori*a: (a) ethyl acetate extract, (b) n-BuOH extract, (c) methanol-chloroform extract, (d) water extract.

**Figure 2.**
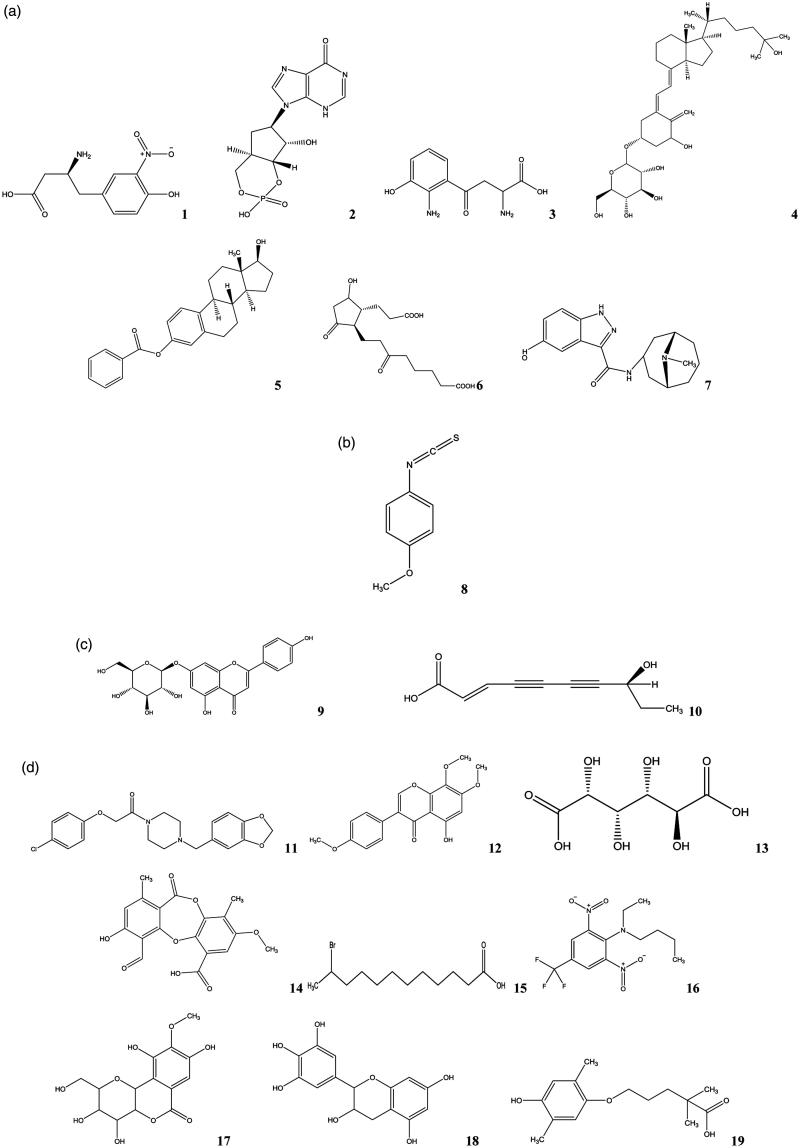
The main compounds from the extracts determined by HPLC/TOF-MS (a) ethyl acetate extract, (b) n-BuOH extract, (c) methanol-chloroform extract, (d) water extract.

HPLC-TOF/MS analysis was done for EA, n-BuOH, Water, and MeCh extracts. The solvent peaks shown in the chromatogram with nineteen natural components were identified by library search and confirmed by mass. The main components in EA extract were determined as 1,25-dihydroxyvitamin-D_3_-glycoside (**4**) and estradiol benzoate (**5**). The other components in EA extract were 3-nitrotyrosine (**1**), 3′,5′-cyclic inosine monophosphate (**2**), 3-hydroxy-dl-kynurenine (**3**), 9,15-dioxo-11*R*-hydroxy-2,3,4,5-tetranor-prostan-1,20-dioic acid (**6**), granisetron metabolite (**7**) (Figures 1(a) and 2(a)). The *n*-BuOH extraction did not give components except compound **8** (4-methoxyphenyl isothiocyanate) as seen in Figures 1(b) and 2(b). MeCh extract gave only two components as apigenin-7-glucoside (**9**) and 8*S*-hydroxy-2-decene-4,6-diynoic acid (**10**) (Figures 1(c) and 2(c)). The water extraction gave the maximum number (9) of components (Figures 1(d) and 2(d)). The components of the water extract were determined as fipexide (**11**), isotectorigenin 7-methyl ether (**12**), D-saccharic acid (**13**), psoromic acid (**14**), 11-bromo-dodecanoic acid (**15**), benfluralin (**16**), bergenin (**17**), epigallocatechin (**18**), 5-(4-hydroxy-2,5-dimethylphenoxy)-2,2-dimethylpentanoic acid (**19**).

For quantitative analysis, 22 standard components were analyzed in the extracts (Table 1). 4-Hydroxybenzoic acid (268.20 mg/kg), apigenin-7-glucoside (133.34 mg/kg), and gallic acid (68.92 mg/kg) were the major components of ethyl acetate extract. The major component of the water extract and BuOH extract was determined as gallic acid (13.98 and 31.12 mg/kg, respectively), while the major component of MeCh extract was determined as apigenin-7-glucoside (13.68 mg/kg). The maximum number of phenolic compounds was determined in EA (18), followed by BuOH (13) and water (9) extracts. All of the phenolic compounds except gentisic acid, chlorogenic acid and catechin were higher amounts in EA extract than the other extracts. On the other hand, naringenin, and protocatechuic acid ethyl ester were not found in all of the extracts. Hashim et al. (1990) found acacetin, luteolin and apigenin glycosides in *Chrozophora* species. Apigenin and quercetin glycosides (Noori 2012) and the acylated flavone glucosides have been reported from *C. tinctoria* (Delazar et al. 2006).

**Table 1.** Quantification of phenolic compounds of the extracts.

	Water	EA	*n*-BuOH	MeCh
Gallic acid	13.98	68.92	31.12	1.08
Caffeic acid	ND	3.91	ND	ND
*p*-Coumaric acid	ND	0.15	ND	ND
Rosmarinic acid	0.20	2.19	0.69	0.09
Chicoric acid	ND	43.93	0.08	ND
Apigenin-7-glucoside	ND	133.34	5.57	13.68
Quercetin	ND	0.44	0.12	ND
Naringenin	ND	ND	ND	ND
Kaempferol	ND	ND	ND	ND
Gentisic acid	2.60	12.51	24.02	ND
4-Hydroxy benzoic acid	0.88	268.20	ND	3.76
Chlorogenic acid	0.67	ND	ND	ND
Vanillic acid	6.44	23.53	3.00	ND
Ferulic acid	ND	43.51	0.35	ND
Salicylic acid	ND	12.33	1.99	ND
Catechin	0.34	0.33	1.02	ND
Protocateuchic acid	ND	3.71	1.62	ND
4-hydroxybenzaldehyde	ND	2.16	ND	ND
Rutin	ND	3.78	2.27	ND
Ellagic acid	0.15	49.59	2.39	ND
Hesperidin	ND	1.88	ND	ND
Protocateuchic acid ethyl ester	ND	ND	ND	ND
Resveratrol	1.46	ND	ND	ND

ND: Not determined.

### Antiproliferative effects of the samples from *C. tinctoria*

**Figure 3.**
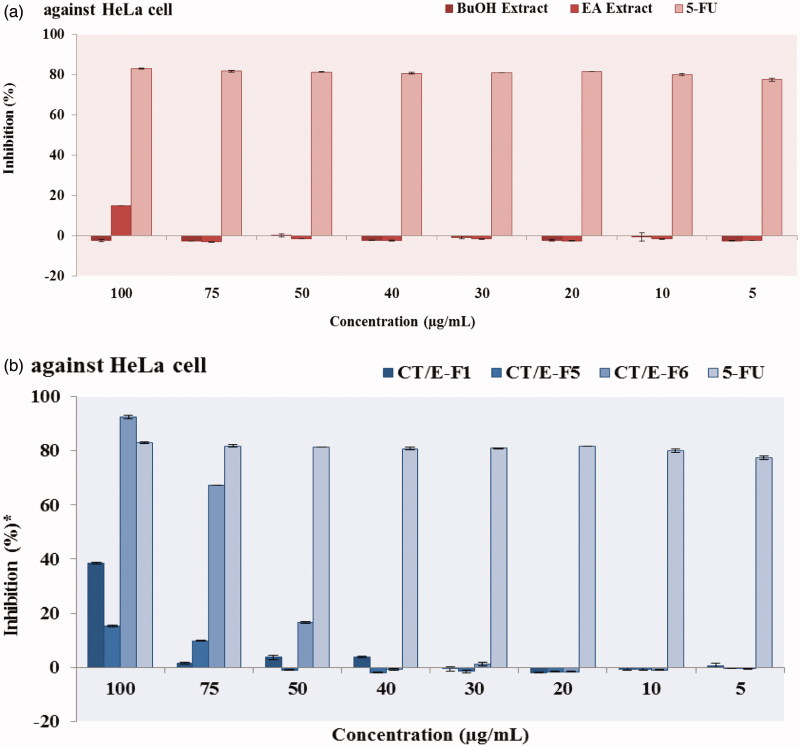
Antiproliferative effects of the ethyl acetate and butanol extracts (a) and active fractions (b) from *C. tinctoria*.

In this study, antiproliferative effects of the samples from *C. tinctoria* were examined on HeLa cell lines at the eight concentrations (5, 10, 20, 30, 40, 50, 75 and 100 μg/mL). EA extract exhibited higher antiproliferative effect than the BuOH extract at the concentration of 100 μg/mL (Figure 3). The potency of inhibitions (at 100 μg/mL) against HeLa cells were: 5-FU > EA extract > BuOH extract.

The antiproliferative effects of EA extract and the fractions that were collected as UV peak from the extract were examined on HeLa cell lines at the eight concentrations (5–100 μg/mL). CT/E-F6 (IC_50_= 64.59 ± 0.01 μg/mL) had higher activity against HeLa cells compared to other fractions, EA extract and 5-FU (standard compound) (Table 2). The potency of inhibition (at 100 μg/mL) against HeLa cells were: CT/E-F6 > 5-FU > CT/E-F1 > CT/E-F5 > EA extract > CT/E-F4 > CT/E-F3 > CT/E-F7 > CT/E-F2 > CT/E-F8.

**Table 2.** The antiproliferative effects of *C. tinctoria* EA extract and obtained fractions at the tested concentrations.

Concentration (μg/mL)	EA extract	CT/E-F1	CT/E-F2	CT/E-F3	CT/E-F4	CT/E-F5	CT/E-F6	CT/E-F7	CT/E-F8	5-FU
100	15.07 ± 0.00	38.47 ± 0.39	−0.43 ± 0.04	9.49 ± 0.66	11.66 ± 0.26	15.32 ± 0.24	92.47 ± 0.71	6.04 ± 0.30	−1.65 ± 0.04	83.20 ± 0.33
75	−3.12 ± 0.22	1.40 ± 0.33	−0.73 ± 0.05	3.58 ± 0.09	−1.24 ± 0.05	9.81 ± 0.05	67.28 ± 0.09	4.43 ± 0.02	−1.72 ± 0.03	81.86 ± 0.43
50	−1.55 ± 0.02	3.64 ± 0.78	−0.33 ± 0.03	0.40 ± 0.05	0.74 ± 0.01	−0.99 ± 0.14	16.56 ± 0.24	−0.92 ± 0.02	−0.28 ± 0.01	81.41 ± 0.13
40	−2.41 ± 0.18	3.93 ± 0.34	−0.46 ± 0.06	0.04 ± 0.00	−1.38 ± 0.04	−1.95 ± 0.12	1.25 ± 0.06	−1.66 ± 0.01	−2.34 ± 0.04	80.82 ± 0.45
30	−1.54 ± 0.16	−0.54 ± 0.05	−0.49 ± 0.02	−0.31 ± 0.01	−0.84 ± 0.02	−1.59 ± 0.05	−0.74 ± 0.01	−1.17 ± 0.03	−0.01 ± 0.02	80.98 ± 0.09
20	−2.65 ± 0.12	−2.03 ± 0.12	−1.41 ± 0.22	−1.47 ± 0.00	−1.39 ± 0.06	−1.67 ± 0.16	−1.63 ± 0.02	−2.21 ± 0.01	−1.17 ± 0.03	81.66 ± 0.07
10	−1.61 ± 0.22	−0.74 ± 0.03	−0.80 ± 0.12	−0.23 ± 0.02	−0.52 ± 0.03	−0.67 ± 0.03	−0.99 ± 0.01	−0.75 ± 0.05	−1.37 ± 0.05	80.16 ± 0.60
5	−2.41 ± 0.06	0.65 ± 0.08	−1.13 ± 0.04	−0.93 ± 0.00	−0.13 ± 0.01	−0.40 ± 0.06	−0.56 ± 0.02	−0.65 ± 0.02	−1.14 ± 0.08	77.58 ± 0.72
	CT/E − F9	CT/E − F10	CT/E − F11	CT/E − F12	CT/E − F13	CT/E − F15	CT/E − F16	CT/E − F17	CT/E − F18	CT/E − F19
100	−1.60 ± 0.14	−1.14 ± 0.15	0.89 ± 0.04	−1.31 ± 0.18	−1.18 ± 0.18	0.84 ± 0.03	−0.14 ± 0.01	−0.24 ± 0.00	6.09 ± 0.05	−0.40 ± 0.01
75	−2.09 ± 0.08	−0.58 ± 0.05	−1.40 ± 0.04	−2.01 ± 0.04	−1.31 ± 0.04	−1.85 ± 0.05	−2.93 ± 0.40	−2.17 ± 0.06	−1.62 ± 0.28	−0.16 ± 0.01
50	−1.54 ± 0.08	−1.09 ± 0.18	−0.27 ± 0.01	−1.55 ± 0.36	−0.81 ± 0.06	−1.80 ± 0.07	−2.22 ± 0.16	−1.43 ± 0.34	−1.96 ± 0.02	−0.40 ± 0.08
40	−2.42 ± 0.07	−1.92 ± 0.32	−1.10 ± 0.03	−1.10 ± 0.28	−1.93 ± 0.08	−2.43 ± 0.06	−2.78 ± 0.24	−2.05 ± 0.24	−1.53 ± 0.48	−0.53 ± 0.04
30	−2.18 ± 0.20	−1.72 ± 0.12	−1.36 ± 0.04	−1.62 ± 0.14	−1.02 ± 0.12	−1.92 ± 0.08	−2.12 ± 0.66	−2.16 ± 0.16	−1.55 ± 0.42	−0.31 ± 0.08
20	−3.22 ± 0.08	−2.26 ± 0.40	−2.39 ± 0.03	−2.71 ± 0.36	−2.09 ± 0.18	−2.50 ± 0.03	−2.47 ± 0.16	−3.15 ± 0.16	−2.67 ± 0.08	−1.26 ± 0.01
10	−0.92 ± 0.07	0.23 ± 0.01	−0.93 ± 0.02	−1.54 ± 0.02	−1.12 ± 0.02	−1.43 ± 0.08	−1.73 ± 0.36	−2.03 ± 0.42	−1.85 ± 0.02	−0.83 ± 0.02
5	−1.54 ± 0.08	0.17 ± 0.02	−1.95 ± 0.03	−2.16 ± 0.66	−1.69 ± 0.05	−1.66 ± 0.05	−1.58 ± 0.06	−1.72 ± 0.18	−1.61 ± 0.14	−0.54 ± 0.06

The antiproliferative effects of EA extract and fractions that did not have a UV peak from the extract, were examined on HeLa cell lines at the eight concentrations (5–100 μg/mL). The potency of inhibitions (at 100 μg/mL) against HeLa cells were: 5-FU > EA extract > CT/E-F18 > CT/E-F11 > CT/E-F15 > CT/E-F16 > CT/E-F17 > CT/E-F19 > CT/E-F10 > CT/E-F13 > CT/E-F12 >CT/E-F9 (Table 2).

Whereas no literature data were available for *C. tinctoria,* other plants of the genus *Chrozophora* were evaluated for their antiproliferative effects on human cell lines. Mothana et al. (2011) investigated the inhibitory effect of the methanol and hot aqueous extracts of *Chrozophora oblongifolia* (Delile) A.Juss. ex Spreng against human urinary bladder carcinoma (5637) and human breast cancer (MCF-7) cell lines. In that study, the hot aqueous extract exhibited moderate effect against 5637 cell line (IC_50 _=_ _298 ± 3 μg/mL).

4-Hydroxybenzoic acid (4-HBA), gallic acid (GA), and apigenin-7-glucoside (A-7-G) were found to be the main components of the EA. Therefore, the antiproliferative effects of these components of *C. tinctoria* were examined on HeLa cell lines. GA exhibited the highest activity against HeLa cells (IC_50 _<_ _5 μg/mL) (Table 4). The potency of inhibitions (at 100 μg/mL) against HeLa cells were found as GA> 5-FU > A-7-G> 4-HBA.

**Table 4.** DPPH radical scavenging and antiproliferative effects of major phenolic compounds of EA extract.

Main phenolic compounds	DPPH radical scavenging IC_50_ (μg/mL)	Antiproliferative effect IC_50_ (μg/mL)
Gallic acid	<5	<5
Apigenin 7-glucoside	134.7 ± 1.2	>100
4-Hydroxybenzoic acid	>150	>100

Some studies have been reported on the antiproliferative effects of the gallic acid and it was found that gallic acid had remarkable effects on some cancer cell lines including HeLa by inducing apoptosis and activating caspases (Ji et al. 2009; You et al. 2010, 2011).

### DPPH radical scavenging activity and total phenolic content of the samples from *C. tinctoria*

**Figure 4.**
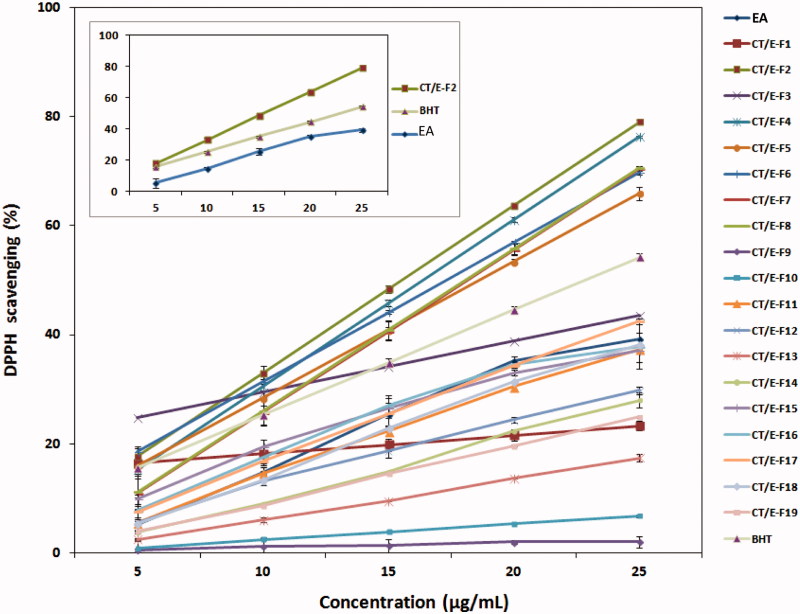
DPPH radical scavenging of the EA extract, fractions, and BHT at the tested concentrations.

DPPH radical scavenging activities of the EA extract, fractions, and BHT are given in Table 3. Lower IC_50_ values indicate higher free radical scavenging activity. The highest free radical scavenging activity was obtained from CT/E-F2 with the lowest IC_50_ value of 14.0 ± 0.0 μg/mL, followed by CT/E-F4 (16.2 ± 0.5 μg/mL) and CT/E-F6 (17.3 ± 0.1 μg/mL). CT/E-F2, CT/E-F4, CT/E-F6, CT/E-F7 (18.6 ± 0.4 μg/mL), CT/E-F8 (17.8 ± 0.4 μg/mL) and CT/E-F5 (18.7 ± 0.0 μg/mL) exhibited higher radical scavenging activity than the reference compound BHT (IC_50_ = 23.1 ± 0.0 μg/mL). Figure 4 shows the dose-response curves of the DPPH radical scavenging activities of the fractions from *C. tinctoria*. DPPH radical scavenging abilities increase with the increased concentration of the samples. IC_50_ value of EA extract was determined as 27.3 ± 0.4 μg/mL. Shahwar et al. (2010) investigated that DPPH radical scavenging activity of the four extracts (pethroleum ether, chloroform, ethyl acetate, methanol/*n*-butanol) from *C. tinctoria* and they observed the best activity in the ethyl acetate extract. This result was similar to that observed in our present study. Sharifi-Rad et al. (2015) observed a noticeable radical scavenging effect in the leaf methanol extract of *C. tinctoria* (IC_50 _= 26.4 ± 0.2 μg/mL).

**Table 3.** Antioxidant activities of the fractions and ethyl acetate extract from C. tinctoria*.

Material	DPPH IC_50_ μg/mL	Total phenolic content μg GAE/mg fraction
EA	27.3 ± 0.4^c^	115.3 ± 0.4^d^
CT/E-F1	63.1 ± 4.2^h^	74.8 ± 3.2^j^
CT/E-F2	14.0 ± 0.0^a^	186.4 ± 1.1^a^
CT/E-F3	31.9 ± 0.3^d,e^	100.7 ± 1.4^f.g^
CT/E-F4	16.2 ± 0.5^a^	138.9 ± 0.7^b^
CT/E-F5	18.7 ± 0.0^a^	125.5 ± 0.5^c^
CT/E-F6	17.3 ± 0.1^a^	126.4 ± 0.4^c^
CT/E-F7	18.6 ± 0.4^a^	126.4 ± 0.4^c^
CT/E-F8	17.8 ± 0.4^a^	127.6 ± 0.5^c^
CT/E-F9	>150	9.2 ± 0.6^l^
CT/E-F10	>150	25.9 ± 0.6^k^
CT/E-F11	32.8 ± 0.7^e^	98.3 ± 0.2^g^
CT/E-F12	43.1 ± 1.8^g^	88.2 ± 0.3^h^
CT/E-F13	61.2 ± 2.3^h^	73.8 ± 0.6^j^
CT/E-F14	39.2 ± 0.8^f^	99.7 ± 0.6^g^
CT/E-F15	32.0 ± 1.4^d,e^	100.9 ± 1.1^g^
CT/E-F16	27.3 ± 2.9^c^	113.2 ± 0.3^d^
CT/E-F17	28.8 ± 0.8^c,d^	108.7 ± 0.2^e^
CT/E-F18	30.8 ± 0.2^d,e^	100.9 ± 0.7^f^
CT/E-F19	48.8 ± 0.4^g^	85.0 ± 0.7^i^
BHT	23.3 ± 0.6^b^	NS

* Values represent averages ± standard deviations for triplicate experiments. Values in the same column with different superscripts are significantly (*p* < 0.01) different, NS: Not Studied.

4-Hydroxybenzoic acid, gallic acid, and apigenin-7-glucoside were the main components of EA. Therefore, the radical scavenging effects of these components were studied at the concentrations of 5-150 μg/mL. DPPH scavenging activity of the tested compounds decreased in the order of gallic acid > apigenin-7-glucoside >4-hydroxybenzoic acid (Table 4). Gallic acid exhibited very strong radical scavenging ability (IC_50 _<_ _5 μg/mL). The radical-scavenging activities of phenolic acids depend on the number of hydroxyl moieties attached to the aromatic ring of the benzoic or cinnamic acid molecule (Karamac et al. 2005).

In the study of Karamac et al. (2005), the radical-scavenging activities of the phenolic acids were found in the order of gallic > gentisic > syringic > caffeic > protocatechuic > sinapic > ferulic > isoferulic > vanillic > *p*-coumaric > *o*-coumaric > *m*-coumaric > salicylic > p-hydroxybenzoic. Gallic acid, with three hydroxyl groups, was observed to be the most active phenolic acid. Zhao et al. (2015) reported that DPPH free radical scavenging activities of some compounds from *Lavandula angustifolia* Mill. (Lamiaceae) and they were determined the IC_50_ value of radical scavenging activity of apigenin 7-*O*-β-d-glucoside as 103.42 μg/mL. In our study, IC_50_ value of apigenin-7-glucoside was determined as 134.7 μg/mL.

The contents of total phenolic compounds in the fractions, expressed as μg gallic acid equivalents (GAE) per milligram of dry fraction, ranged between 9.17 ± 0.64 to 186.37 ± 1.10 μg/mg. CT/E-F2 had the highest total phenolic content (186.37 ± 1.10 μg/mg). In our study, the total phenolic content of EA extract was determined as 115.3 ± 0.4 μg/mg. Shahwar et al. (2010) determined that the total phenolic content of the ethyl acetate extract of *C. tinctoria* was 353.5 ± 5.5 mg GAE/g of crude extract. In this study, a significant (*p* < 0.01) correlation was observed between total phenolic content and DPPH scavenging indicating that phenolic compounds may be primarily responsible for radical scavenging activity. DPPH radical scavenging activity of stem and leaf extracts from *C. tinctoria* was studied by Sharifi-Rad et al. (2015) and they reported that the leaf extracts exhibited higher activity than stem extracts due to the phenolic concentration of leaf extracts was higher than that of stem extracts. Similarly, in our study, we found significant correlation between total phenolic content and radical scavenging activity.

## Conclusions

In this study, we have successfully determined the biologically active fractions (CT/E-F2 and CT/E-F6) from ethyl acetate extract of *C. tinctoria.* Their high antiproliferative and antioxidant effects may be attributed to the presence of gallic acid. The results showed that *C. tinctoria* could be used as a natural source in food, cosmetics and pharmaceuticals industries. Further studies are needed to isolate and identify the compounds from these active fractions and also to evaluate *in vivo* biological activities of the isolated compounds.
